# Nexus of regional integration, socioeconomic determinants and sustainable development in belt and road initiative countries

**DOI:** 10.1371/journal.pone.0254298

**Published:** 2021-07-09

**Authors:** Atta Ullah, Chen Pinglu, Saif Ullah, Shujahat Haider Hashmi

**Affiliations:** 1 School of Management, Huazhong University of Science and Technology (HUST), Wuhan, China; 2 Faculty of Management Sciences, SZABIST, Karachi, Pakistan; 3 School of Economics, Huazhong University of Science and Technology (HUST), Wuhan, China; University of Western Australia, AUSTRALIA

## Abstract

This study evaluates the nexus of regional integration, socioeconomic determinants and sustainable development (SD) by investigating the effect of health, humans and age structure on sustainable development, with the regional integration (RI) as the moderating variable. Socioeconomic determinants have an important role in sustainable development, while regional integration has fueled up the development process. The sample is based on 64 Belt and Road (BRI) countries from 2003–2018. Pair-wise correlation results indicate that human development, health expenditure and age structure showed a positive relationship with sustainable development. Two-step System-GMM direct effect outcomes are mixed and reveal that human development, health expenditure per capita, age structure, governance index and population size have a positive impact on sustainable development. On the other hand, e-government, government size, and globalization showed negative effects on SD. Apart from that, the moderating channel of regional integration (RI), interaction term with human development and health expenditure, showed a significant and positive impact on sustainable development. However, age structure interaction with regional integration showed a negative impact on SD. Other socio-economic factors, i.e., governance index and population contribute positively towards SD. It can be concluded that the dynamic nature of sustainable development is positive and the net present value is increasing. Therefore, BRI countries are on the sustainable path from 2003–2018, as suggested by economic and social welfare theory. The integration of BRI can be labeled as an entrance to successful sustainable development. However, weak e-government systems, globalization and government resources need to be utilized amicably in Belt and Road countries. Driscoll-Kraay standard-errors regression confirmed and validated the two-step System-GMM results. The findings of the current research have important policy implications for balanced and sustainable growth.

## 1. Introduction

Today, brilliant minds all over the world are discussing an important question, i.e., how the goal of sustainable development can be achieved in a country. Sustainable development is defined as a fluid activity directed at stabilizing the present and future challenging requirements [1]. For sustainable development, health is both an outcome and a resource. Countries with deficiency and a high prevalence of diseases cannot achieve sustainable development goals [2, 3]. Policymakers emphasize raising human capital development, because it is considered an essential tool to improve public health, enhance welfare systems, equalities and poverty alleviation, which lead towards achieving sustainable development. However, this overall process requires significant expenditure, especially on health, to improve the health sector [4].

Worldwide, each year, catastrophic health expenditure is faced by 150 million people, which underlines the need for designing a health system that offers protection for financial risk for surgery [5]. Zhang, Rahman [6] emphasized the indispensability of universal health coverage (UHC), being a global health priority, for sustainable development, as it ensures the provision of superior health facilities to all citizens whenever needed, without financial risk. WHO (World Health Organization) focuses on the improvements to health financing reforms, health-service coverage, and providing hundred percent safety from deprivation for the entire population of the country, regardless of gender, residential location and economic status. Recently, De Ceukelaire and Bodini [7] have highlighted the need for addressing public health systems as an integral part of public health initiatives beyond self-governing boundaries to affect worldwide geo-economics.

The development of human capital is important for long-term sustainable growth and improves the well-being and dignity of a human being’s life. Djuric and Filipovic [8] have expressed concern over the human and social capital management and sustainable development based on the complexity theory, arguing that human and social capital may present a virtual platform and contribute to sustainable development. According to Chams and Garcia-Blandon [9], sustainable development goals (SDGs) can be achieved through human capital and with new techniques of integration.

Protection of human beings and quality of life depend on several elements including health advancement, social, economic, and environmental development. In most of the countries, average life expectancy is increasing, which is a healthy sign for the community. However, the age structure share/age dependency ratio is also increasing rapidly, and is one of the most potent transformative forces to affect society and the sustainable development path [10]. The ageing structure trend has created social and economic sustainability concerns in communities worldwide, as people tend to live longer because of medical advances and other factors like technology. The population in the developed and emerging economies would rise significantly in the coming decades, as projected by WHO, predicting a significant rise in the population dependency ratio. Therefore, a sustainable atmosphere must be established for the ageing population structure and the possibilities of complementing principles explored to enhance sustainable development aspects [11].

The basic principle of Sustainable Development (SD) is to make a country economically efficient, a social welfare state, and environment-friendly. Regional integration is growing and can facilitate sustainable development in the region, working as a facilitator, particularly in growing markets, for trade advancement, and socio-economic growth. Brautigam and Tang [12] claimed that the millennium century is, by all means, the century of China, that showed the world the country’s ability for unprecedented progress. The issue of sustainable development has been addressed by China in the whole Eurasia through the BRI proposition as the key to achieve the mutual objectives. Taking advantage of the essence of the initial Silk Road, China recognized the BRI framework as the network supporter for the BRI. It is thus an attempt to accelerate the integration in the market place of Eurasia that will turn the trade channel originally driven by efficient trade on the Chinese Silk Road between the Mediterranean Sea and China in about 114 BCE. China’s modern Silk Road style is mainly defined as the land-based “Silk Road Economic Belt” and “21^st^ Century Maritime Silk Road”. According to Tambo, Khayeka-Wandabwa [13], China’s BRI involves more than 65 countries. It is estimated to exceed a budget of USD 1trillion to link China across Asia, Europe, Middle East, and Africa directly and indirectly. The impacts of BRI on global development can be categorized into (a) information harnessing, technology, and health, (b) ease in commerce and trade, (c) augmented energy resource safety, and (d) progress towards planetary human-driven development.

Governance and sustainable development are mutually linked in this globalized world. Good governance practices facilitate global and domestic investors to make their investment decisions freely, leading to sustainable development [7]. Moreover,e-government focuses on sustainable development (SD) and is contemplated as a technology by governance that should upgrade institutional work and reorganize the interactivity network among governments and citizens, among businesses and employees, and governments and their constituents, if the technology-adoption situation improves in a country [14].

Relatively less research has been carried out on the determinants of sustainable development. Therefore, the current empirical literature on national saving rates studied by Hess [15] can be considered first. In a study of Malaysia, Pardi, Salleh [16] found that the emphasis on economic growth had led to a new notion and dimensions of sustainable development. Kaimuri and Kosimbei [17] too observed the need for a global paradigm shift to sustainable development to address various problems facing regions and countries. Qian, Ho [18] lamented the lack of research on the relationship between age structuring and sustainable development. Grazuleviciute-Vileniske, Seduikyte [11] posited that at present, individual welfare becomes sustainable development and a human- centered approach; therefore, population and associated issues such as health expenditure per capita must be viewed from the perspective of sustainable development, based on social, economic, and environmental sustainability.

The recent global pandemic has affected every field of life, increased the per capita health expenditure of every country, and raised a question mark over every country’s sustainable development. Therefore, it is vital to study sustainable development and its socio-economic determinants to cope with the pandemic and future global pandemics, through robust public health systems beyond self-governing boundaries to affect worldwide geo-economics [7]. Hickel [19] expressed concern that human development (HD) does not pay heed to environmental sustainability, considering the close ties between wealth and environmental impacts.

Eryılmaz, Bakır [20] emphasize the need for an efficient regional system to help achieve sustainable development. The regional integration link of BRI to sustainable development has not been explored much [21]. China’s issue of sustainable economic development has been addressed in Eurasia and it is argued that the BRI proposition is the key to achieving the mutual objective of sustainable development [13]. Abbas, Gillani [22] recommend studying sustainable development determinants concerning regional integration and governance, e-government and globalization factors. Moreover, Naeher and Narayanan [23] have pointed out that sustainability has been attracting unprecedented attention in recent years, in developing countries. Moreover, with the changing degrees of success and the recent global protectionism, policymakers persistently argued that deeper regional integration establishes an environment conducive to sustainable development, security, and peace. Many international development organizations have aimed at enhancing regional integration. Still, very little empirical evidence is observed that permits policymakers to measure and match integration levels across different regions/ sub-regions to map accomplished progress against stated goals.

Sustainable development is a combination of economic, social, and environmental progress; under the premise, it is essential to incorporate and settle the economic, social, and environmental features within an all-inclusive and stable sustainable development framework. The beneficial influence of sustainability on social factors can be achieved only when the development is spread all over the population [24]. Social and governance factors and reporting of these factors were encouraged by regulators of different countries, including the Belt and Road Initiative countries. However, sustainable development is a new concept that is not adequately explored in One Belt countries at the regional level. On the other side, practitioners and researchers believe that e-government is an essential instrument that can equip administrators with the opportunity to alleviate social and economic unfairness and boost sustainable development and prosperity at the national level [14]. Therefore, it is a novel concept and noteworthy to recognize sustainable development and its socio-economic determinants within the Belt and Road Initiative (BRI) regional integration, because BRI is aimed at enhancing cooperation and promoting integration to improve policy coordination, ease connectivity, attain unhindered trade and development, realize regional integration, and strengthen attachment among the peoples.

The current study explores the dynamic effect of human development, age structure, and health expenditure on sustainable development, with the moderating effect of regional integration among Belt and Road countries, along with other socio-economic factors such as governance index, e-government development index, population size, government size, and globalization index, from 2003 to 2018.

The current study contributes in different ways to the novel concept and body of knowledge with an untapped set of variables such as adjusted net savings, including particulate emission damage index of the World Bank for sustainable development, health expenditure, human development, age structure. It also emphasizes other socio-economic factors like governance, e-government, globalization etc., concerned with moderating the regional integration of Belt and Road countries by employing the latest empirical two-step system GMM technique validation through D-K standard error regression. Findings reveal that sustainable development estimated through extended Solow economic growth was positive in both ways (direct and moderating channel) as suggested by economic theory, and the social welfare net present value was increasing; therefore, BRI sample countries are on a sustainable development path. Findings indicate that BRI countries are fulfilling the current requirement without harming future needs; however, initiative countries need to improve and implement effective regional integration systems, practices, and e-government systems to maintain sustainable growth and benefit from the regional integration under BRI. This is because human development, better health and age structuring atmosphere, technology transformation, globalized resources, and good governance are related to the sustainable development of a country. Currently, BRI is contributing to achieving sustainable development and can be more effective if efficiently utilized at its full potential in terms of multi-dimensional regional integration and globalized resources. BRI multi-dimensional regional integration can be labeled as the doorway to increasing social, economic, and environmental (energy) happenings as a passage of success and sustainable development.

The remaining study is organized as follows. Literature review and hypothesis development are discussed in next section. The third section elaborates data and methods concerning the theoretical framework and empirical modeling. The next section provides baseline analysis and model findings, with detailed discussion. Concluding remarks are provided at the end, with the study limitations and suggested direction of future research.

## 2. Literature review

Sustainable development (SD) refers to meeting the requirements of the current generation by providing a quality life without compromising the requirements of future generations [25]. Sustainable development has been established from social, environmental, and economic fields to attain the world’s institutional, political, technological, and societal requisites.The combination of social, economic, and environmental factors is necessary because as per the development theory, all the activities are aimed at securing a balance among the commercial, social, and environmental dimensions of the sustainable development process.

The variables used in this study to determine the relationship between sustainable development and its socio-economic determinants with regional integration as moderating variable are: human development, health expenditure per capita, age structure share, governance composite, e-government, globalization index, population size, and government size, based on past studies [4, 10, 13, 15–17, 22, 26–30].

### 2.1 Human development index and sustainable development

Human development encompasses dimensions such as enabling a person to govern, health, life expectancy, education (i.e., mean years of schooling and expected years of schooling), and per capita for the standard of stable living. Human development that lacks sustainability and empowerment is challenging to lead. Human development promotes sustainable development and global integration [31] and focuses on health, knowledge, and well-being of a sustainable society [32].

Human development is based on a healthy and long life, knowledge, and a decent standard of living for assessing progress in the long term. Study findings showed that human development has a positive and significant relationship with sustainable development [26]. Hess [15] studied the determinants of sustainable development by taking HDI as an independent determinant and adjusted net saving dependent proxy for the sustainable development by extending the Solow growth model. He stated that the ambiguity in the indication over the initial Human Development Index (HDI) implies a measure for convergence theory–if upheld then, this variable’s effect on economic growth will be negative. However, as measured by the index’s life expectancy and literacy components, the initial levels of human resources can be closely linked to subsequent economic development. Hess (15) found that HDI has a positive impact on sustainable development (i.e., adjusted net savings) and sustainable policies.

Another study by Boyacıoglu [3] in Turkey investigated the relationship between health indicators and sustainable development comparing it with other countries for 1980 and 2008, with variables including GDP (per capita), and human development, i.e., including birth, life expectancy, rate of infant mortality and health measures. The result demonstrates that the mortality rate decreased and life expectancy rose significantly by an increase in Turkey’s health expenditure, which is also favorable to human development. Chikalipah and Makina [33] showed that human development and sustainable growth are co-integrated. Improvement in human capital level makes a society sustainable in terms of development, as suggested by the social welfare theory.

Based on the above, we frame the first hypothesis as follows:

*H*_*1*_: *Human development has a significant and positive impact on sustainable development*.

### 2.2 Age structure and sustainable development

Age structure share is defined as the population age groups: the first category consists of children and adolescents under 15 dependent on their parents or other family members. The second group is the working-age population between 16 and 64 years, considered an important determinant contributing to the sustainable development of a country. The third group consists of people who depend on their savings in their elderly age, at 65 years or more. Everybody needs to retain their current living standards after retirement or during old age from a social perspective, although this would not be feasible, given the current savings rates. As a result, much of the population requires government assistance through a safety net that places a burden on future generations. The nexus between ageing structure and sustainability has been studied by Dietz, Neumayer [34], who found significant positive support of age structure share between 16 to 64 for sustainable development.

Hess [15] confirmed that understanding the age structure share of the group between 16 and 64 as a determinant is helpful for sustainable development (adjusted net savings rate) policies. He found that a fraction of the labor-age population positively influences the adjusted net saving rate, which is the proxy of sustainable development. The initial distribution of social capital is calculated by the 25-year-old population’s average education years. The population’s age structure, expressed in dependency pressures, can also affect the capacity to save from a given national income. An increase in the ratio of the population under 15 (youth dependents) to the total population, or to the population aged 15 to 64 (net producers), will continue to demand a higher proportion of income for the children’s current social welfare expenditure (education, healthcare, food and clothing), which is counted as consumption expenditure in estimating the national income. In line with the life cycle theory of consumption, an increase in the proportion of the population which is 65 or more (older dependents) will also help reduce the national saving rate, as a higher proportion of the population moves into the dissolving years and with increasing elderly healthcare expenses. The youth and elderly burdens of dependency tend to be reversed, with the former declining during the fertility transition and the latter rising with a replacement fertility approach. A significant decline may occur several decades later, after the onset of fertility decline, when a nation experiences a ’demographic dividend’ from a rising labor force age. Considering the above discussion, the average percentage of the population aged 15–64 over the period (APL) was used to capture the population’s age structure as a determinant, as suggested by [10, 15, 35], because it is important to consider the age structure in the BRI countries.

Boyacıoglu [3] concluded that the population’s age structure is reflected in the dependence burdens and may also affect the ability to save from a given national income. Qian, Ho [18] studied the aging-friendly and economic, environmental, and social sustainability relationship and found a significant sustainability linkage. Nilsson [10] posited that sustainable work life is important to all age groups, especially when working life extended to a higher age. Therefore, it is important to consider and make a sustainable work-life balance in most countries, due to demographic age structure dependency challenges. Consistent with the life-cycle theory of intake as a higher population proportion moving into years of breakdown and increasing healthcare costs for elderly persons, the current study postulates the following hypothesis based on the findings of the [15]:

*H*_*2*_: *Share of the population of age 15 to 65 has a significant*, *positive impact on sustainable development*.

### 2.3 Health expenditure and sustainable development

A country’s health-care system indicates and better represents its sustainability. Health expenditure refers to the medical and non-medical costs to facilitate and provide basic health care services to the population. Health care is the global priority and key to the Sustainable Development Goals (SDGs) defined by World Health Organization to ensure the provision of health care to all citizens on the basis of equality and safety [6]. It is thus an important determinant of a nation’s sustainable development.

The relationship between health expenditure and sustainable development was investigated by Boyacıoglu [3] within the economic and health indicators in Turkey between 1980 and 2008 by comparing them with other countries. Results demonstrated that increase in health expenditure has a significant positive influence on sustainable development in Turkey. Boos and Holm-Muller [36] carried out a cross-country analysis to determine sustainable development, using a genuine savings index (GSI). They used analogous regressions to the work to carry out the analysis. They concluded that a shrinking genuine saving is a sign of erosion of sustainable development stock, which can serve as an economic warning to a country. However, they failed to include human capital, health expenditure, income distribution, inequality, age structure share of year 18–64, and per-capita income as determinants which are essential factors to determine sustainable development.

Today, experts generally believe that global warming is a reality. Global warming effects and the resilience of vulnerable populations thereto vary widely, but overall global warming overlaps current vulnerabilities. Global warming will further affect poor people’s health, restrict access to drinking water and pose a real threat to food security and sustainability in many African, Asian, and Latin American countries [37]. Therefore, it is important to explore health and sustainable development-related concepts, because lack of basic human needs for proper life applies to poverty and income inequality; when people cannot have enough food to survive, are unable to go to school, or do not have enough access to health services, they may be considered to be in poverty, regardless of their wealth [38].

Cheung and Padieu [39] studied the heterogeneity effect of NCMS (New Cooperative Medical Scheme) on family savings in China’s rural area across different income groups in which NCMS proposed to deal with rustic inhabitants. The set of demographic, socio-economic, and geographical determinants of savings with instrumental variables were analyzed through OLS regression. Their findings showed that implementing health caution schemes appears to be a suitable approach to economic growth, by reducing savings and boosting consumption. The effects of public health expenditure on health outcomes in Ghana were investigated by Kofi Boachie, Ramu [4], in which human spending per capita, savings, and other health factors were estimated. Their results showed that public health expenditures are essential for improving health conditions, as suggested by the social welfare theory. Another study conducted by Sahnoun [40] examined the health spending relationship with economic development (growth) for forty-four years by using non-stationary time series econometrics and confirmed that a positive relationship exists between health expenditure and economic development (growth), which is important for sustainable development.

Kaitelidou, Galanis [41] explored the utilization of health facilities in Greece and found that disparities in people’s ability to access and utilize health care could be reduced through an increase in health care expenditure, which leads to sustainable development as suggested by the economic and social welfare theory. Leon, Jimenez [42] examined the social movement’s content and impact, directed by the National Health Forum, and their role in the development of the National Public Health System for the period 2009 to 2018. They found that the National Health Forum, as a People’s Health Movement, played a central role in strengthening the health system and sustainable development.

Accordingly, the third hypothesis is framed as follows:

*H*_*3*_: *Health expenditure per capita has a positive influence on sustainable development*.

### 2.4 Regional integration and sustainable development

Regional integration is a recently emerging phenomenon, especially after the Belt and Road initiative and plays a vital role because more than 65 countries are integrated based on the regionalism and development theory for the common objective of social, economic, and environmental dimensions of sustainable development. The growing incorporation of national economies through BRI in the world offers a concurrent example. It indicates that regional integration can function as a facilitator in multi-dimensional ways, particularly in trade advancement, digital transformation, growing markets, regional action, and the growth of sustainable socio-economic factors. Yu and Chang [21] posited that the BRI initiative, based on the regional integration and development theory, is directed at improving global collaboration and encouraging connectivity to achieve sustainable growth and strengthen regional attachment; such measures could promote human development and health-care by creating better employment opportunities to save money for old-age benefits.

Based on the regionalization and development theory, the effect of regional integration on economic development was investigated by Obere, Muthoga [43], who employed the generalized method of moments (GMM) and found that economic development was significantly stimulated by regional integration. Therefore, the phenomena of regionalization have a potential relationship with the development of a sustainable economy. Brautigam and Tang [12] claimed that China had addressed the issue of sustainable economic development by offering the BRI proposition of sustainable development phenomenon as the key to achieving the common objectives of human development, health care and economic growth through trade, connectivity, investment and infrastructure development. Wang and Selina [44] argued that BRI is likely to practice a development theory process somewhat as a moderator to its counterparts worldwide. If bilateral cooperation is positive, it may mitigate political instability and enhance local development with regional progression [45]. The proposed hypothesis is as follows:

*H*_*4*_: *There is a significant moderating influence of regional integration between sustainable development and its determinants*.

### 2.5 Other socio-economic factors

The current study includes other critical socio-economic determinants that can influence sustainable development and with regional integration, like governance composite, e-government development index (EGDI), government size, population size, and globalization index. Rajkumar and Swaroop [46] showed that countries with good governance positively impact sustainable development. Corruption too affects sustainable development. Stojanovic, Ateljevic [30] investigated good governance effect for countries at different levels on specific sustainable development indicators, particularly socio-economic development. Their findings reveal the positive effects of good governance dimensions on sustainable development; their direction and intensity are statistically significant, though weak governance impacted negatively. However, they reported quite heterogeneous effects with regard to particular dimensions of sustainable development (social, economic and environmental) across different countries.

The word e-government is a relatively modernized terminology, and is considered a vital tool for modernizing government in the 21^st^ century. In this modern era, every country’s internet usage has increased due to a technology- friendly environment. However, people wish for ease of life in e-service from the government, leading to a country’s sustainable development [47]. According to Shaw, Kim [48], e-government depends on innovative and advanced technologies with long-term effects. However, currently, the e-government system of developing countries is not up-to-date and useful, which needs to be addressed. Most of the countries included in the BRI are at the developing stage and many follow the manual system; however, recently, many countries have focused more on technology-based governance.

Different aspects of socio-economic factors have emerged due to the theory of globalization. Based on this milieu, the paradigm of globalization is similar to the theory of the word-system [49]. Globalization has a mixed impact on socio-economic development that varies across regions and countries [14]. Moreover, globalization can affect the relationship by making financial investments in the green economy and environment-friendly technology, as environmental sustainability is the primary concern of modern society, leading to sustainable development [50].

Besides, in recent decades, high population growth has caused a significant breakdown of development prospects, as confirmed and reported by [51]. Guney [27] examined the impact of population growth on the sustainable development of 146 countries for the period 1990–2012 by using GLS (generalized least squares), and confirmed that the impact of population on sustainable development varies with the level of economic development; it has a positive effect in developed countries and negative effect in developing countries. Another control factor, total government spending, is included as the indicator of government size. The relationship of government size with economic development was examined by Asimakopoulos and Karavias [52], who identified the optimum stage of government size by applying the generalized method of moments (GMM) and general non-linear panel approach. Their findings demonstrate that the total effect of government expenditure on economic progress is uneven at above or below the optimal level in developing and developed countries. Oladele, Mah [53] examined the role of government spending size on sustainable growth from 1980–2014 in South Africa, finding the existence of a long-run association between government spending and economic development; both factors are positively correlated in the long-run, but negatively in the short run, which shows that expenditures matter for sustainable development.

## 3. Data and methodology

### 3.1 Data and measurement of variables

The quantitative research method is adopted to test data from 2003 to 2018 empirically for sixty-four Belt and Road initiative countries. In areas such as finance, macroeconomics, and international and regional studies, it is now customary to work with panel data to back policy decisions by empirical research using time series and panel data. Panel data popularity is also due to important recent developments in analyzing it with software i.e., Stata, to carry out sophisticated computations [54]. This study uses a panel dataset because of its greater variability and efficiency. Through the panel, the dataset can detect and measure accurate statistical effects, which other approaches cannot [55]. Hence panel data methods improve the efficiency of econometric estimates, and are more suitable for determining the factors affecting sustainable development, because our panel data set includes a large sample of 64 countries over 16 years. Thus, the advantage of panel data is their ability to control for individual heterogeneity and better ability to study the dynamics of adjustment, which entails more variability and less collinearity among the variables [56].

Moreover, suitable and more appropriate methods like the two-step system generalized methods of moments (GMM) and Driscoll-Kraay (D-K) standard-error regression ensure accurate inferences and control the auto-correlation by taking the lagged dependent variables and removing the endogeneity issue, omitted variables bias, unattended panel heterogeneity and measurement errors [57]. Endogeneity is a major issue in wider panel data as in BRI countries in our case. Therefore, the utilization of the panel data method for analysis by employing the two-step system GMM and robust check D-K regression is useful. The sample countries are listed in *“Appendix A”* in S1 Appendix. Many sustainable development indicators have emerged over the years, due to the failure to use GDP and income as reliable indicators of sustainable development. Strezov, Evans [58] determined different indicators for calculating sustainable development, including EF (Ecological Footprint), ESI (Environmental Sustainability Index), EPI (Environmental Performance Index), CWI (Change in Wealth Index), GSI (Genuine Savings Index), GWI (Global Well-Being Index), HPI (Happy Planet Index), and SSI (Sustainable Society Index). The indices were analyzed for their capability to determine the environmental, economic, and social dimensions of sustainable development. SSI and GSI (Two indices) measures are more comprehensive, as they are based on three sustainable development dimensions, while other indices were limited to economic and environmental dimensions or socio-economic, socio-environmental dimensions alone.

SSI (Sustainable Society Index) data is available only till 2014. However, the Genuine Savings Index (GSI) is based on adjusted net saving and is the most appropriate, comprehensive and updated index proposed by the World Bank in the 1990s. This index is related to three dimensions, viz., social, economic, and environmental factors, and has been recently updated in 2020, with its data available till 2018. Adjusted net saving is formally known as genuine saving, consisting of social, economic, and environmental factors. For measurement of dependent variable-sustainable development, we used the index of adjusted net saving, which has been extensively accepted as an inclusive measure based on all three dimensions of sustainable development [15–17, 27, 28, 59–62].

Adjusted net saving, including certain omissions damage, is the updated figure and, based on all these, sustainable social, economic, and environmental dimensions which measure saving in a broad context, including physical capital, natural capital, social capital, knowledge stock, and economically worthiness. Therefore, Pearce and Atkinson [63] were the first to introduce adjusted net saving (ANS) as a sustainability indicator, based on the Hartwick Rule’s reformation. However, recently, World Bank proposed a compressive index of adjusted net saving for sustainable development, updated based on the Changing Wealth of Nations 2018 for building a sustainable future [64]. Recently, Koirala and Pradhan [28] endorsed using the Genuine Savings Index (GSI) based on the adjusted net saving rate index of the World Bank.

The intendent variables are human development, age structure share and health expenditure per capita. Regional Integration is the moderating variable and for each year observation, RI is ranked throughout a period that ranges from 0 to 1 in scale and followed from [65]. While governance principle component analysis index, e-government development index, government size, population size, and globalization index are the control factors. The governance index includes six indicators of development: the rule of law, control of corruption, regulatory quality, political stability and absence of terrorism, government effectiveness, and voice and accountability [16, 30, 66]. Also, globalization index based social, political, and economic dimensions of globalization concerned on 0 to 100 points of higher values denote greater globalization and proxy recommended [67, 68]. The E-government development index (EGDI) comprises a wide-ranging investigation of 193 countries of United Nations (UN) having an online existence that evaluates their e-government plans and strategies, national-websites, and how they are working in particular and common sectors to provide vital services [47]. The detailed measurement and data description is reported in “*Appendix B”* in S1 Appendix.

### 3.2 Model framework

**Fig 1:**
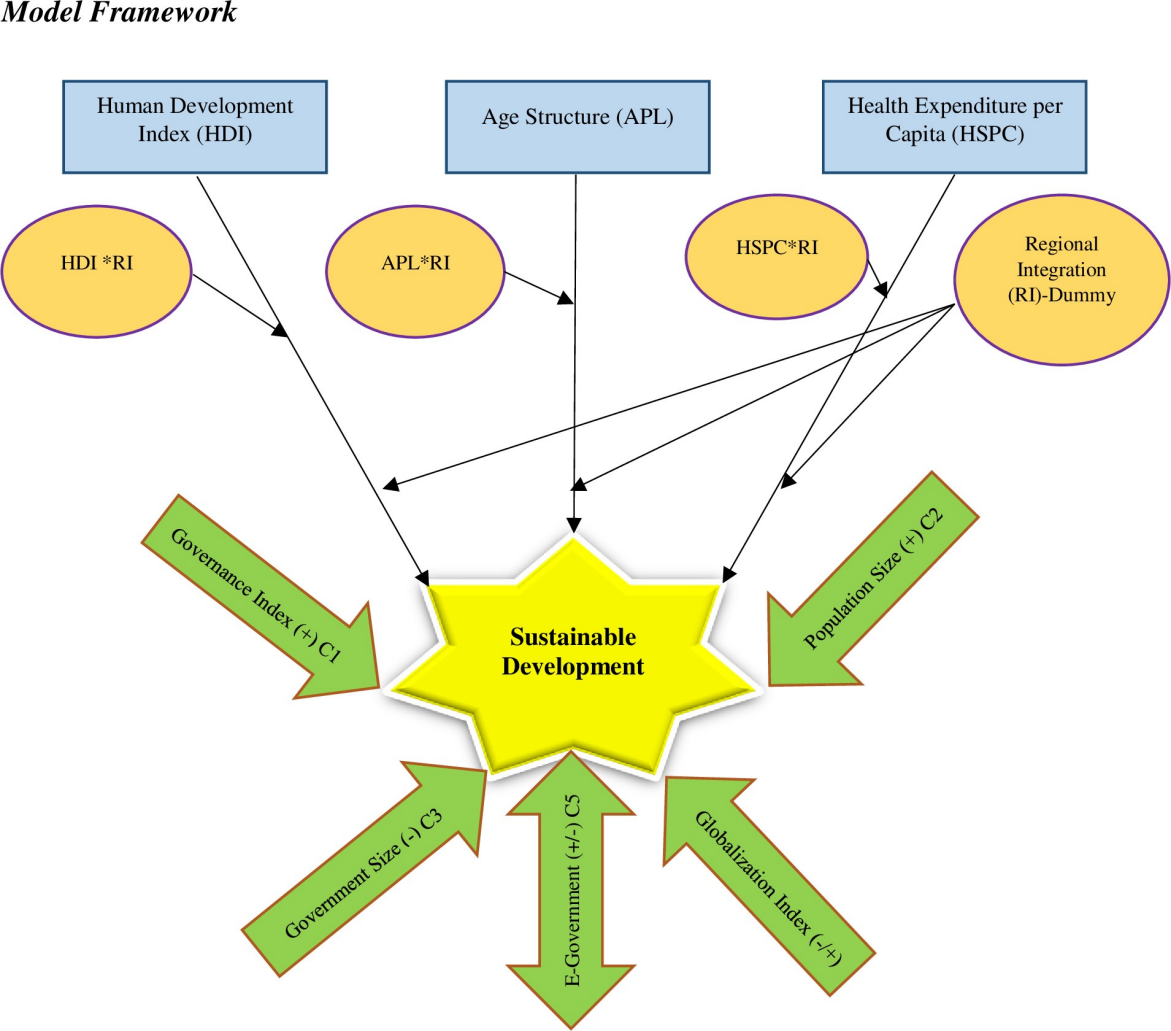
Conceptual framework.

Based on the theory and past literature, the framework of this study is stated in Fig 1.

The theoretical framework is based on the extended version of the Solow standard economic growth model. By following the Cobb–Douglas function of country’s production output at the national level and in accordance with the studies of Hess [15], Pardi, Salleh [16] and Koirala and Pradhan [28], the theoretical model can be written as follows:

$$\frac{\text{sy}}{\text{y}}=\Phi +\alpha \;\left(\frac{\text{dy}*}{\text{y}*}\right)-\beta \text{n}+\gamma \omega +\text{p}$$ (1)

where, Eq (1) rate of economic growth is defined as being directly related to the rate of net savings (s). However, the rate of economic growth would be negatively linked to the natural resources depletion, the rate (ω), and rate of population growth (p) because the proposed model of saving includes the depletion of natural resources reflecting the way of reduction of natural capital may affect the saving levels of future generations, concerning the sustainable development model. Besides, y indicates the real national output, output per capita detonated by y*, and labor force growth rate by (n). Input shares are *α*, *β*, and *γ*. Φ is equal to g +*α* f + *β* m + γh and reflects all the associated factors, including the rate of growth. Production factor’s growth rates K (capital), L (labor), and R (natural resources) are represented by (f, m, and h), and the technological progress rate is denoted by (g). Progress in capital quality is supposed to be positive (f > 0), which signifies the modern technologies used for the latest capital products. Simultaneously, labor quality improvement is also supposed to be positive (m > 0) and would be reflected in the formation of human capital with investments in health, education, and nutrition.

The relationship to determine the sustainable development (adjusted net saving) and its determinants concerning the model described earlier can be written by following earlier studies [16, 28, 69]. In Eq (2), SD represents the dependent variable-sustainable development, HDI represents the human development index, APL indicates age structure and HSPC represents the human expenditure as independent determinants. RI represents the moderating variable of regional integration. Moreover, control factors include the governance index (GOV), e-government index (EGDI), government size (GS), population size (PS), and globalization index (GI).


$$\text{SD}\left(\text{ANSR}\right)=\int \left(\text{HDI},\;\text{APL},\;\text{HSPC},\;\text{RI},\;\text{GOV},\;\text{EGDI},\;\text{GS},\;\text{PS},\;\text{GI}\right)+\varphi_{t}+\varepsilon_{\text{it}}$$ (2)

### 3.3 Empirical estimation–two-step system GMM and Driscoll-Kraay standard errors

Generalized methods of moments (GMM) provide better results with correct model specifications than single-equation models and techniques. Two-step system GMM is the best suitable, where we do not know the distribution of the dependent variable [70]. The lag-value of SD is taken in GMM to make it a dynamic model and avoid the issue of autocorrelation as prevails in the static regression model. Therefore, GMM provides more efficient and accurate estimates by controlling the lag-effect of its own dependent variable-SD to predict the future more accurately in the long term. Thus, previous studies of Arellano and Bond [71], Arellano and Bover [72], Blundell and Bond [73], Roodman [74], Farhadi, Islam [75], Arminen and Menegaki [76], Lin [77], Ahmad, Khattak [78], Abbas, Gillani [22], and Ullah, Kui [24] relied on and preferred this technique for the panel dataset. To deal with the potential endogeneity issues, the GMM estimator is best suited. The econometric feature of the two-step system GMM includes both OLS and 2SLS, where 2SLS indicates a special case of two-step system GMM. The augmented estimator is the system GMM [79].

Two-step GMM (Arellano-Bond have both one and two-step variants)—are more efficient because they are robust to heteroscedasticity and autocorrelation. Two-step System GMM is good when N (number of cross-sections) is greater than T (period). This study has N = 6 and T = 16. Therefore, it is an excellent case to use the GMM approach proposed by [71, 74]. Farhadi, Islam [75] and Ahmad, Khattak [79] prefer reducing the difference and two-system GMM because of the best alternative internal instruments.

The results of two-step system GMM are also validated through Driscoll-Kraay standard-errors regression, because this approach yields robust standard errors by correcting the problems of heteroscedasticity, cross sectional dependence and auto-correlation presence, as suggested by [80, 81]. Therefore, the DK regression endorses the earlier findings of the two-step system GMM and serves as a suitable alternate robust method.

The functional form of the two-step system GMM is expressed as

$${\text{y}}_{\text{it}}={\text{X}}_{\text{it}}\;\beta +\vartheta \gamma_{\text{i},\text{t}-1}+\varphi_{t}+\varepsilon_{\text{it}}$$ (3)

Arminen and Menegaki [76] and Ahmad, Khattak [78] posited that the cross-sectional units are denoted by subscript i (here in our sample, 64 countries) while time is indicated by t (here in our sample period of 16 years). Following several authors [76, 78, 82] year dummies represent *φ*_*t*_ which control for common shocks such as the global financial crises of 2007–2009. It is assumed that the error term is composed of the fixed individual effect ε holding the properties as follows: E [*c*_*i*_] = E [ε_*it*_] = [*c*_*i*_*ε*_*it*_] = 0, and reflecting idiosyncratic shocks; an attempt was made to eliminate the individual fixed effects by taking the difference of Eq (3). Hence, this condition can be written as

$$\Delta \gamma_{\text{it}}=\Delta {\text{X}}_{\text{it}}\;\beta +\vartheta \Delta \gamma_{\text{i,t-1}}+\varphi_{t}+\varepsilon_{\text{i}}$$ (4)

where Δ is the differenced operator in Eq (4).

By following the earlier mentioned equations and studies of Abbas, Gillani [22], Koirala and Pradhan [28], Ahmad, Khattak [78], Arminen and Menegaki [76], Lin [77], Waisman, Ye [69] and Pardi, Salleh [16], the econometric model can be written as follows;

The direct-channel static econometric model can be written as follows:

$${\text{SD}}_{\text{i},\text{t}}=\alpha_{0}+\beta_{1}{\left(\text{HD}\right)}_{\text{i},\text{t}}+\beta_{2}{\left(\text{APL}\right)}_{\text{i},\text{t}}+\beta_{3}{\left(\text{HSPC}\right)}_{\text{i},\text{t}}+\beta_{4}\left(\text{Control}\;\text{Factors}\right)+\varphi_{t}+\varepsilon_{\text{it}}$$ (5)

The direct-channel dynamic econometric model of two-step system GMM is explained as follows:

$${\text{SD}}_{\text{i},\text{t}}=\alpha_{0}+\beta_{1}{\text{SD}}_{\text{i},\text{t}-1}+\beta_{2}{\left(\text{HD}\right)}_{\text{i},\text{t}}+\beta_{3}{\left(\text{APL}\right)}_{\text{i},\text{t}}+\beta_{4}{\left(\text{HSPC}\right)}_{\text{i},\text{t}}+\beta_{5}\left(\text{Control}\;\text{Factors}\right)+\varphi_{t}+\varepsilon_{\text{it}}$$ (6)

The interaction term of regional integration was used to moderate the relationship between the determinants and sustainable development (ANS). The model specifications make use of these interaction terms (determinants of SD*RI) by following the work of Lin [77], Brambor, Clark [83], and Brambor, Clark [84]. Static and dynamic models of two-step System GMM, the interaction term of HDI*RI, APL*RI, and HSPC*RI are reported as follows in Eqs 7–12.

An econometric Interaction term of human development and regional integration in static model can be written as follows:

$${\text{SD}}_{\text{i},\text{t}}=\alpha_{0}+\beta_{1}{\left(\text{HD}\right)}_{\text{i},\text{t}}+\beta_{2}{\left(\text{APL}\right)}_{\text{i},\text{t}}+\beta_{3}{\left(\text{HSPC}\right)}_{\text{i},\text{t}}+\beta_{4}{\left(\text{RI}\right)}_{\text{i},\text{t}}+\beta_{5}{\left(\text{HD}*\text{RI}\right)}_{\text{i},\text{t}}+\beta_{6}\left(\text{Control}\;\text{Factors}\right)+\varphi_{t}+\varepsilon_{\text{it}}$$ (7)

Interaction term of human development and regional integration in dynamic model of two-step system GMM can be explained as follows:

$${\text{SD}}_{\text{i},\text{t}}=\alpha_{0}+\beta_{1}{\left(\text{SD}\right)}_{\text{i},\text{t}-1}+\beta_{2}{\left(\text{HDI}\right)}_{\text{i},\text{t}}+\beta_{3}{\left(\text{APL}\right)}_{\text{i},\text{t}}+\beta_{4}{\left(\text{HSPC}\right)}_{\text{i},\text{t}}+\beta_{5}{\left(\text{RI}\right)}_{\text{i},\text{t}}+\beta_{6}{\left(\text{HDI}*\text{RI}\right)}_{\text{i}.\text{t}}+\beta_{7}\left(\text{Control}\;\text{Factors}\right)+\varphi_{t}+\varepsilon_{\text{it}}$$ (8)

An econometric interaction term of age structure share and regional integration in static model can be written as follows:

$${\text{SD}}_{\text{i},\text{t}}=\alpha_{0}+\beta_{1}{\left(\text{HDI}\right)}_{\text{i},\text{t}}+\beta_{2}{\left(\text{APL}\right)}_{\text{i},\text{t}}+\beta_{3}{\left(\text{HSPC}\right)}_{\text{i},\text{t}}+\beta_{4}{\left(\text{RI}\right)}_{\text{i},\text{t}}+\beta_{5}{\left(\text{APL}*\text{RI}\right)}_{\text{i},\text{t}}+\beta_{6}\left(\text{Control}\;\text{Factors}\right)+\varphi_{t}+\varepsilon_{\text{it}}$$ (9)

Interaction term of age structure share and regional integration in dynamic model of two-step system GMM can be explained as follows:

$${\text{SD}}_{\text{i},\text{t}}=\alpha_{0}+\beta_{1}{\left(\text{SD}\right)}_{\text{i},\text{t}-1}+\beta_{2}{\left(\text{HDI}\right)}_{\text{i},\text{t}}+\beta_{3}{\left(\text{APL}\right)}_{\text{i},\text{t}}+\beta_{4}{\left(\text{HSPC}\right)}_{\text{i},\text{t}}+\beta_{5}{\left(\text{RI}\right)}_{\text{i},\text{t}}+\beta_{6}{\left(\text{APL}*\text{RI}\right)}_{\text{i}.\text{t}}+\beta_{7}\left(\text{Control}\;\text{Factors}\right)+\varphi_{t}+\varepsilon_{\text{it}}$$ (10)

An econometric interaction term of health expenditure per capita and regional integration in static model can be written as follows:

$${\text{SD}}_{\text{i},\text{t}}=\alpha_{0}+\beta_{1}{\left(\text{HDI}\right)}_{\text{i},\text{t}}+\beta_{2}{\left(\text{APL}\right)}_{\text{i},\text{t}}+\beta_{3}{\left(\text{HSPC}\right)}_{\text{i},\text{t}}+\beta_{4}{\left(\text{RI}\right)}_{\text{i},\text{t}}+\beta_{5}{\left(\text{HSPC}*\text{RI}\right)}_{\text{i},\text{t}}+\beta_{6}\left(\text{Control}\;\text{Factors}\right)+\varphi_{t}+\varepsilon_{\text{it}}$$ (11)

Interaction term of health expenditure per capita and regional integration in dynamic model of two-step system GMM can be explained as follows:

$${\text{SD}}_{\text{i},\text{t}}=\alpha_{0}+\beta_{1}{\left(\text{SD}\right)}_{\text{i},\text{t}-1}+\beta_{2}{\left(\text{HDI}\right)}_{\text{i},\text{t}}+\beta_{3}{\left(\text{APL}\right)}_{\text{i},\text{t}}+\beta_{4}{\left(\text{HSPC}\right)}_{\text{i},\text{t}}+\beta_{5}{\left(\text{RI}\right)}_{\text{i},\text{t}}+\beta_{6}{\left(\text{HSPC}*\text{RI}\right)}_{\text{i}.\text{t}}+\beta_{7}\left(\text{Control}\;\text{Factors}\right)+\varphi_{t}+\varepsilon_{\text{it}}$$ (12)

where, SD(ANSR) is the adjusted net saving rate, which is the proxy of sustainable development, HD represents human development, APL indicates age structure share, HSPC represents the health expenditure per capita, and RI indicates regional integration. Further, control factors include the governance composite, e-government index, population size, government size, and globalization index.

## 4. Results and discussion

### 4.1 Stationary (unit root) and westerlund cointegration test

For checking the stationary of the sample data of years 2003 to 2018, unit root tests of Levin, Lin & Chu t* of 2002, Im, Pesaran and Shin W-stat of 1997, ADF—Fisher Chi-square of 1999 and PP—Fisher Chi-square test of 2001 were applied. The unit root results reported that all the variables are stationary at level [I (0)] except population size, which is stationary at first difference [I (1)] in Levin, Lin & Chu test. Further, the Westerlund test for co-integration of 2007 was conducted to check the long-term relationship among the variables considered; the results show that there exists a long-term co-integrating relationship among sustainable development and its determinants and we reject the null hypothesis (H_0_) of no co-integration at a 1% significance level. Detailed results of unit root and co-integration tests are reported in “*Appendix C”* in S1 Appendix.

### 4.2 Summary statistic

Table 1 provides descriptive statistics and Table 2 shows the pair-wise relationship of sustainable development with its determinants. Descriptive statistics exhibit the average values of variables, and the variability of data values is within the range. The results depict that human development, age structure share (APL), health expenditure per capita (HSPC), governance composite, e-government development index (EGDI) and population size (PS) have a statistically positive and significant relationship, at 14.5%, 32.25%, 17%, 25%, 9.8%, and 18% respectively, with sustainable development at a 1% significance level. However, the globalization index is negatively (-1.1%) correlated with sustainable development, but government size is relatively more negative (-9.6%) at 1% level of significance. Regional integration has a (-3.6%) negative association with sustainable development. The interaction terms of regional integration and independent variables, i.e., HSPC*RI, show a positive (8.4%) relation, while HD*RI and APL*RI indicate (1.2%) a (1.1%) negative relationship with sustainable development.

**Table 1:** Descriptive statistics.

Variables	Obs.	Mean	Std. Dev.	Min	Max
SD	1024	10.734	11.937	-37.41	44.191
HD	1024	.694	.138	.303	.935
APL	1024	65.72	6.483	49.437	86.398
HSPC	1024	422.607	509.92	.01	2837.14
Governance	1024	0	1	-2.03	3.706
EGDI	1024	.48	.167	.093	.908
GS	1024	15.199	4.686	3.46	30
PS	1024	69.581	224.56	.35	1392.73
GI	1024	63.586	12.481	26.24	86.15
RI	1024	.375	.484	0	1
HD*RI	1024	.271	.358	0	.935
APL*RI	1024	24.847	32.322	0	85.975
HSPC*RI	1024	190.312	433.524	0	2837.14

**Table 2:** Pairwise correlations.

Variables		(1)	(2)	(3)	(4)	(5)	(6)	(7)	(8)	(9)	(10)	(11)	(12)	(13)
(1)	SD	1.000												
(2)	HD	0.145***	1.000											
(3)	APL	0.322***	0.535***	1.000										
(4)	HSPC	0.170***	0.407***	0.475***	1.000									
(5)	Governance	0.250***	0.468***	0.396***	0.495***	1.000								
(6)	EGDI	0.098***	0.593***	0.459***	0.496***	0.462***	1.000							
(7)	GS	-0.096***	0.345***	0.169***	0.413***	0.322***	0.285***	1.000						
(8)	PS	0.180***	-0.103***	0.051*	-0.152***	-0.103***	-0.042	-0.187***	1.000					
(9)	GI	-0.011	0.424***	0.354***	0.455***	0.486***	0.440***	0.338***	-0.062**	1.000				
(10)	RI	-0.036	0.158***	0.064**	0.129***	0.056*	0.261***	0.063**	0.013	0.175***	1.000			
(11)	HDI*RI	-0.012	0.281***	0.154***	0.236***	0.112***	0.383***	0.100***	-0.000	0.273***	0.375***	1.000		
(12)	APL*RI	-0.011	0.204***	0.134***	0.165***	0.080**	0.305***	0.073**	0.017	0.205***	0.393***	0.487***	1.000	
(13)	HSPC*RI	0.084***	0.426***	0.252***	0.450***	0.261***	0.507***	0.235***	-0.069**	0.398***	0.567***	0.582***	0.506***	1.000

*Note*. *** p<0.01,

** p<0.05,

* p<0.1 indicate significance at 1%, 5%, and 10% levels, respectively.

### 4.3 Results of two-step system generalized method of moments (gmm)–direct channel

Two-step system GMM dynamic models keep changing concerning time, whereas static models are at equilibrium in a steady state. Table 3, as seen below, computes outputs for column (1) and (2) for a static model (two-step difference GMM method), whereas, columns (3), (4), and (5) provide estimates for the dynamic model (two-step system GMM). Direct-channel results depict that Arellano–Bond (AR) test, applied for the zero autocorrelation in first-differenced, reveals AR (1) showing the existence of first-order autocorrelation. AR (2) shows no second-order autocorrelation, implying that the moment conditions are correctly specified and the original error term is serially uncorrelated at second-order. The Hansen and Sargan test was applied to examine the instrument’s reliability and control the over-identifying restrictions in the analysis. Therefore Hansen test and Sargan tests to estimate the other restrictions were also validated [24, 70]. The number of instruments (J-statistics) employed is 51, which indicates that the two-step system GMM is a valid instrument for this study. The Wald-CHI Square test indicates that variables used in the model are significant. Overall authentication and specifications confirmed the appropriateness of the two-step system GMM model [22, 74, 82].

**Table 3:** Results of direct impact- two-step system generalized methods of moment (GMM).

Variables	(1)	(2)	(3)	(4)	(5)
	Static Model		Dynamic Model		
	OLS	Fixed Effect	OLS	Fixed Effect	Two-step System GMM
	SD	SD	SD	SD	SD
L. Sustainable Development (SD)			0.909***	0.615***	0.904***
			(0.012)	(0.024)	(0.012)
Human Development (HD)	4.814	24.072	5.670**	21.696**	4.503**
	(7.078)	(24.761)	(2.868)	(8.483)	(2.247)
Age Structure Share (18–64 Years)	0.659***	-0.202	0.019	-0.253***	0.003
	(0.084)	(0.231)	(0.034)	(0.090)	(0.022)
Health Expenditure Per Capita (HSPC)	0.006***	0.001	0.001	-0.001	0.001***
	(0.001)	(0.001)	(0.000)	(0.001)	(0.000)
Governance Composite	3.220***	0.655	0.247	0.935*	0.177**
	(0.388)	(1.362)	(0.159)	(0.516)	(0.264)
E-Government	-6.566	-11.679*	-3.089	-7.100***	-2.839***
	(4.772)	(5.997)	(1.905)	(2.341)	(0.558)
Government Size	-0.439***	-0.712***	-0.091***	-0.387***	-0.084***
	(0.078)	(0.245)	(0.031)	(0.068)	(0.030)
Population Size	0.009***	-0.006	0.001	-0.009	0.001***
	(0.001)	(0.021)	(0.001)	(0.013)	(0.000)
Globalization Index	-0.389***	-0.018	-0.041*	-0.113*	-0.028
	(0.052)	(0.145)	(0.021)	(0.064)	(0.017)
Constant	-4.500	24.795	1.068	23.422***	2.047*
	(4.452)	(16.926)	(1.778)	(5.567)	(1.107)
Observations	1,024	1,024	960	960	960
R-squared	0.277	0.087	0.892	0.487	
Year Dummies	Yes	Yes	Yes	Yes	Yes
System GMM Post Analysis					
AR1	-3.594				
AR1 p-value	0.000326				
AR2	1.993				
AR 2 p-value	0.110				
Sargan Test	587.5				
Hansen Test	61.02				
Hansen (p-value)	0.17				
No. of Instruments J-Stat	51				
Wald test-CHI2	70934				
Wald test-CHI2 p-value	0				
No. of Groups	64				

*Note*. Standard errors in parentheses,

*** p<0.01,

** p<0.05,

* p<0.1 indicate significance at 1%, 5%, and 10% levels, respectively, Used Stata outreg2, xtabond2 command—[74].

Outcomes in Table 3 revealed the impact of various determinants on SD based on the static regression model and dynamic model of system GMM. Based on the results, Wald test validates the two-step system GMM results, as reported in column (5); these findings imply that our dynamic model controls omitted variable bias, unobserved panel heterogeneity, and measurement errors. The results reveal that the lag-value of sustainable development (i.e., dependent variable) has a coefficient value [0.904], with a p-value less than 1%, which signifies the dynamic nature of sustainable development. Human development has a positive and statistically significant impact on sustainable development at a 5% significance level with the coefficient value of 4.503, indicating that a 1% increase in human development level would lead to a 4.503% improvement in sustainable development. The age structure (share of average ages 15–64 years) has a positive but insignificant impact on sustainable development with a coefficient value of 0.003. On average, a 1% variation in age structure raises sustainable development by 0.3% over the given time. Besides, health expenditure shows a statistically positive impact, at 1% significance level with a coefficient value of 0.001 on sustainable development. It indicates that a one percent increase in per capita health expenditure level would cause a 0.1% improvement in sustainable development in BRI countries. Other socio-economic factors, i.e., the governance index and population size, show a positive and significant impact on sustainable development. In contrast, e-government development and government size have a negative but significant impact on sustainable development. Further, the globalization index negatively influences the sustainable development path in BRI economies. The detailed results are reported in Table 3.

### 4.4 Regional integration results

The results of two-step system GMM model, including the interaction terms of intended determinants with moderating variable-regional integration (RI) i.e., HD*RI, APL*RI, and HPSC*RI for sustainable development, are demonstrated in Tables 4–6. Roodman [74] accepted standards of its asymptotical effectiveness, and the xtabond2 command was followed. Column (1), based on pooled OLS in Tables 4–6 provides estimates of interaction terms, and column (2) exhibits the results based on panel fixed effect. The results of the dynamic model are demonstrated in column (3) based on pooled OLS, in column (4) using panel fixed effect, and in column (5) based on the final model of two-step system GMM. Outcomes in Tables 4–6 reveal that AR (1) term is significant, which means first-order autocorrelation exists in the data, while AR (2) becomes statistically insignificant (AR (2) p-value is > 0.05), which indicates that no autocorrelation or serial correlation in the second order. The results of two-step system GMM method reported in Colum (5) of these tables provide J-statistic values = 54, 52 and 58, respectively. In the Hansen and Sargan’s test, over-identifying restrictions are valid, which supports the instrument reliability and fails to reject the null hypothesis. Overall, estimated models are up to the mark to ensure the accurate inference and validation of results, which indicates that the two-step system GMM is an estimation technique appropriate for the current study, as in this study, number of N (64) is more than the number of T (16).

**Table 4:** Interaction term effect of HD*RI on SD.

Variables	(1)	(2)	(3)	(4)	(5)
	Static Model		Dynamic Model		
	OLS	Fixed Effect	OLS	Fixed Effect	Two-step System GMM
	SD	SD	SD	SD	SD
L. Sustainable Development (SD)			0.909***	0.614***	0.868***
			(0.012)	(0.024)	(0.012)
Human Development (HD)	4.274	36.102	5.281*	26.596***	12.001**
	(7.172)	(26.348)	(2.903)	(9.812)	(5.432)
Age Structure Share (18–64 Years)	0.658***	-0.250	0.014	-0.269***	0.013
	(0.085)	(0.257)	(0.034)	(0.097)	(0.023)
Health Expenditure Per Capita (HSPC)	0.006***	0.001	0.001	-0.001	-0.001***
	(0.001)	(0.001)	(0.000)	(0.001)	(0.000)
Governance Composite	3.218***	0.715	0.241	0.953*	0.970***
	(0.389)	(1.374)	(0.159)	(0.519)	(0.177)
E-Government	-6.299	-9.945*	-2.121	-6.585***	-2.817**
	(5.082)	(5.879)	(2.021)	(2.535)	(1.154)
Government Size	-0.436***	-0.685***	-0.088***	-0.378***	-0.159***
	(0.078)	(0.245)	(0.031)	(0.068)	(0.041)
Population Size	0.009***	-0.006	0.001	-0.008	0.001***
	(0.001)	(0.021)	(0.001)	(0.013)	(0.000)
Globalization Index	-0.388***	-0.027	-0.043**	-0.115*	-0.090***
	(0.052)	(0.146)	(0.021)	(0.064)	(0.031)
Regional Integration (RI)	-1.387	-1.263	-0.615	-0.583	-2.241***
	(3.686)	(3.264)	(1.441)	(1.629)	(0.481)
HD*RI	1.519*	0.269	0.115**	0.277	2.393***
	(5.190)	(4.602)	(2.028)	(2.212)	(0.623)
Constant	-4.147	19.311	1.512	20.969***	2.369
	(4.502)	(17.610)	(1.802)	(6.478)	(1.441)
Observations	1,024	1,024	960	960	960
R-squared	0.278	0.092	0.893	0.488	
Year Dummies	Yes	Yes	Yes	Yes	Yes
System GMM Post Analysis					
AR1	-3.603				
AR1 p-value	0.000315				
AR2	1.951				
AR 2 p-value	0.153				
Sargan Test	318.9				
Hansen Test	60.09				
Hansen(p-value) Test	0.219				
No. of Instruments/J-Stat	54				
Wald test-CHI2	61537				
Wald test-CHI2 p-value	0				
No. of BRI Countries	64				

*Note*. Standard errors in parentheses,

*** p<0.01,

** p<0.05,

* p<0.1 indicate significance at 1%, 5%, and 10% levels, respectively, Used Stata xtabond2 command—[74].

**Table 5:** Interaction term effect of APL*RI on SD.

Variables	(1)	(2)	(3)	(4)	(5)
	Static Model		Dynamic Model		
	OLS	Fixed Effect	OLS	Fixed Effect	Two-step System GMM
	SD	SD	SD	SD	SD
L. Sustainable Development (SD)			0.910***	0.613***	0.865***
			(0.012)	(0.024)	(0.013)
Human Development (HD)	4.086	31.661	4.529	23.994**	12.044**
	(7.213)	(28.148)	(2.908)	(10.070)	(5.404)
Age Structure Share (18–64 Years) APL	0.674***	-0.239	0.044	-0.266***	0.018
	(0.096)	(0.242)	(0.039)	(0.093)	(0.028)
Health Expenditure Per Capita (HSPC)	0.006***	0.002	0.001	-0.001	-0.001***
	(0.001)	(0.001)	(0.000)	(0.001)	(0.000)
Governance Composite	3.206***	0.618	0.227	0.881*	0.949***
	(0.389)	(1.430)	(0.159)	(0.522)	(0.165)
E-Government	-5.636	-9.056	-1.564	-6.030**	-1.496
	(5.019)	(6.059)	(1.995)	(2.475)	(1.167)
Government Size	-0.437***	-0.671***	-0.087***	-0.371***	-0.163***
	(0.078)	(0.244)	(0.031)	(0.069)	(0.041)
Population Size	0.009***	-0.005	0.001	-0.008	0.001***
	(0.001)	(0.021)	(0.001)	(0.013)	(0.000)
Globalization Index	-0.392***	-0.016	-0.046**	-0.107*	-0.102***
	(0.052)	(0.146)	(0.021)	(0.065)	(0.031)
Regional Integration (RI)	2.337	3.231	3.809	2.024	0.323
	(7.036)	(6.587)	(2.744)	(2.798)	(1.313)
APL*RI	-0.041	-0.065	-0.066	-0.036	-0.014
	(0.106)	(0.097)	(0.041)	(0.042)	(0.019)
Constant	-5.138	20.093	-0.021	21.575***	2.172
	(5.033)	(16.601)	(2.027)	(5.966)	(1.369)
Observations	1,024	1,024	960	960	960
R-squared	0.278	0.093	0.893	0.488	
Year Dummies	Yes	Yes	Yes	Yes	Yes
System GMM Post Analysis					
AR1	-3.604				
AR1 p-value	0.000314				
AR2	1.940				
AR 2 p-value	0.124				
Sargan Test	320.1				
Hansen Test	59.65				
Hansen(p-value) Test	0.19				
No. of Instruments J-Stat	52				
Wald test-CHI2	66163				
Wald test-CHI2 p-value	0				
No. of BRI Countries	64				

*Note*. Standard errors in parentheses,

*** p<0.01,

** p<0.05,

* p<0.1 indicate significance at 1%, 5%, and 10% levels, respectively, Used Stata xtabond2 command—[74].

**Table 6:** Interaction term effect of Health Expenditure*RI on SD.

Variables	(1)	(2)	(3)	(4)	(5)
	Static Model		Dynamic Model		
	OLS	Fixed Effect	OLS	Fixed Effect	Two-step System GMM
	SD	SD	SD	SD	SD
L. Sustainable Development (SD)			0.910***	0.615***	0.871***
			(0.012)	(0.024)	(0.014)
Human Development (HD)	4.589	37.296	4.529	29.917***	12.223**
	(7.139)	(26.324)	(2.908)	(9.961)	(6.085)
Age Structure Share (18–64 Years)	0.656***	-0.243	0.044	-0.244***	-0.004
	(0.085)	(0.250)	(0.039)	(0.094)	(0.022)
Health Expenditure Per Capita (HSPC)	0.006***	0.001	0.001	-0.002*	-0.001***
	(0.001)	(0.002)	(0.000)	(0.001)	(0.000)
Governance Composite	3.214***	0.747	0.227	1.050**	1.074***
	(0.389)	(1.402)	(0.159)	(0.521)	(0.206)
E-Government	-5.985	-10.047*	-1.564	-6.995***	-2.541**
	(4.988)	(5.891)	(1.995)	(2.440)	(1.092)
Government Size	-0.437***	-0.688***	-0.087***	-0.387***	-0.152***
	(0.078)	(0.245)	(0.031)	(0.069)	(0.049)
Population Size	0.009***	-0.006	0.001	-0.008	0.001***
	(0.001)	(0.021)	(0.001)	(0.013)	(0.000)
Globalization Index	-0.390***	-0.027	-0.046**	-0.114*	-0.088**
	(0.052)	(0.147)	(0.021)	(0.064)	(0.035)
Regional Integration (RI)	-0.352	-1.249	3.809	-0.818*	-0.897***
	(0.892)	(1.047)	(2.744)	(0.480)	(0.109)
HSPC*RI	0.000	0.000	0.001	0.001	0.001***
	(0.001)	(0.001)	(0.001)	(0.001)	(0.000)
Constant	-4.282	18.256	-0.021	17.615***	3.114**
	(4.497)	(17.474)	(2.027)	(6.447)	(1.484)
Observations	1,024	1,024	960	960	960
R-squared	0.277	0.092	0.893	0.489	
Year Dummies	Yes	Yes	Yes	Yes	Yes
System GMM Post Analysis					
AR1	-3.611				
AR1 p-value	0.000305				
AR2	1.957				
AR 2 p-value	0.104				
Sargan Test	318.6				
Hansen Test	59.13				
Hansen(p-value) Test	0.16				
No. of Instruments J-Stat	58				
Wald test-CHI2	46979				
Wald test-CHI2 p-value	0				
No. of BRI Countries	64				

*Note*. Standard errors in parentheses,

*** p<0.01,

** p<0.05,

* p<0.1 indicate significance at 1%, 5%, and 10% levels, respectively, Used Stata xtabond2 command—[74].

The empirical outcomes in column (5) of Table 4, based on the two-step system GMM results, confirm the dynamic nature of sustainable (i.e., dependent variables) with the coefficient value of 0.868 with p-value less than 1%. Moreover, outcomes show that the interaction term of HD*RI has a positive effect on sustainable development with coefficient value of 2.393, which is significant at less than 1% level. It implies that a one percent increase in the interaction of HD*RI leads to a 2.393% improvement in sustainable development. The results show that HD has a statistically significant impact on sustainable development at a significance level of less than 1%. The summary results of other variables in Table 4 indicate that share of average ages (15–64 years) has a significant impact on sustainable development. Governance index and population size have a positive impact, at a significance level of less than 1%, on sustainable development. Per capita health expenditure, government size, and globalization index document a statistically significant but negative impact on sustainable development at a significance level of less than 1%. E-government development has a negative effect on sustainable development at a 5% significance level, indicating that the e-government system in BRI needs further improvement. The detailed results are reported in Table 4.

In Table 5, the two-step system GMM results in column (5) reveal that the sustainable development (i.e., dependent variable) lag coefficient is [0.865], with a p-value less than 1%, which signifies the dynamic nature of the sustainable development path in BRI countries. The share of average ages 15–64 years indicates that the age dependency ratio for the old age or retirement savings disposal contributes positively to sustainable development. The moderating variable of regional integration shows a positive but insignificant impact on sustainable development. However, the interaction term of age structure share*regional integration indicates a slightly negative effect. The age structure share 16–64 years is predicted to be optimistic; however, results indicate that it is not effective yet at the regional level, which needs to be focused on.

Moreover, the interaction results of APL*RI indicate that HD contributes positively to sustainable development at less than 5% significance level. Governance composite and population size have a statistically positive impact on sustainable development at a less than 1% significance level. However, health expenditure per capita, government size, and globalization index show a statistically significant but negative effect on sustainable development. Moreover, e-government development has a statistically negative effect on sustainable development, which indicates a weak e-government system in BRI countries, requiring more focus on e-system practices. In Table 5, detailed results of age structure share and regional integration are provided.

In Table 6, the empirical outcomes of the two-step system GMM in column (5) examines the interaction effect of health expenditure per capita and regional integration. The dynamic model results show that the lag-variable of sustainable development (i.e., dependent variable) has a coefficient value of 0.871, with a p-value less than 1%, which signifies the dynamic nature of sustainable development and its positive effect in BRI countries from 2003 to 2018.The interaction term of health expenditure with regional integration (HSPC*RI documents a significant positive effect on sustainable development with the coefficient value of 0.001, at a 1% significance level. It indicates that a 1% variation in the interaction effect of HSPC and RI would cause a 0.01% increase in sustainable development in BRI countries.

Moreover, results depicted that governance composite and population size have a statistically positive and significant impact on sustainable development at a 1% significance level. In contrast, HD contributes positively to sustainable development at a 5% significance. The share of average ages 15–64 years indicates a negative impact when we employed HSPC*RI interaction term. Further, EGDI and globalization have a statistically negative but significant impact on sustainable development, which suggests that the e-government system in BRI and utilization of globalization resources are not optimally deployed and require further modification and improvement. The detailed results are shown in Table 6.

### 4.5 Driscoll-Kraay standard errors regression robustness check

Next, we apply DK regression as an additional robustness measure and report the results of the direct and indirect models. The detailed results of the direct channel are reported in “*Appendix D”* in S1 Appendix, columns 1 and 2, while the robustness results of the indirect channels are reported in *“Appendix E”* in S1 Appendix columns 1 to 6. Overall results of D-K regression validate and confirm the findings of the static models, pooled OLS and Fixed effect methods. The D-K approach yields robust standard errors by correcting the problems of heteroscedasticity, cross-sectional dependence and auto-correlation presence, as suggested by Driscoll et al. and Dar et al. [80, 81]. Therefore, this method endorses the prior findings of the two-step system GMM. These results further validate the outcomes of two-step System GMM, which is best in examining the endogeneity biases, omitted variables, over identifying restriction, measurement errors, and the autocorrelation in the panel dataset. Further, System GMM is efficient because of its robustness to heteroscedasticity and autocorrelation; the econometric trick of the two-step system GMM includes the best case of OLS, fixed effect and 2SLS [22, 74, 82].

### 4.6 Discussion and comparison of results

The current study documents the results of dynamic model OLS, fixed effect, and two-step system GMM in Table 3, and outcomes of interaction terms in Tables 4–6. Economic theory assumes that if net savings are positive, the net existing social welfare value would increase, which means that the potential profit value could exceed the current cost value. Conversely, a persistently negative adjusted net saving implies an economy on an unsustainable path. Therefore, the findings of system GMM reveal that the BRI countries are on a sustainable path concerning regional integration interactions, by increasing social welfare health benefits. Further, overall findings were validated by DK standard-error regression. These results are aligned with the studies of Hess [15], based on 52 developing countries from 2001 to 2006, Pardi, Salleh [16] in Malaysia, Kaimuri and Kosimbei [17] in Kenya and Koirala and Pradhan [28] based on 12 developing Asian countries, who investigated the determinants of sustainable development and argue that a country is on a sustainable development path if adjusted net savings are positive. Therefore, our study findings support the economic and development theory and Hess [15], Pardi, Salleh [16], Kaimuri and Kosimbei [17] and Koirala and Pradhan [28] outcomes.

In the current study, overall results of endogenous and exogenous variables contribute to mixed findings based on 1024 observations from the sample of 64 BRI countries. Correlation of all independent variables HD (14.5%), APL (32.25%), and HSPC (17%) was positive with sustainable development (Adjusted net savings) at the significance level. Simultaneously, the correlation of other socio-economic control factors, governance index (25%), e-government development index (9.6%), and population size (18%) has a positive relationship with sustainable development. This indicates that HD, APL, HSPC, Governance composite, EGDI, and PS contributed positively to a sustainable path, while government size and globalization index have a negative association with the sustainable development of sample of BRI countries. The interaction term of HSPC*RI shows a positive relationship with sustainable development, while HD*RI and APL*RI have a negative relationship.

Two-step system GMM dynamic model is a valid and appropriate approach, with the support of accepted standards of J-statistics, Arellano–Bond (AR) test, Hansen test, Sargan test, and Wald-CHI test, as stated by several authors [22, 74–78]. Two-step system GMM final model is reported in column 5. The outcomes in Table 3 reveal that HD has a positive and significant impact on sustainable development at a 5% significance level. Likewise, the HD*RI interaction term in Table 4 showed a positive impact on sustainable development at a 1% significance level. Therefore, the first research hypothesis of our study stands proved that HD has a positive effect on SD, which endorses the findings of Gnegne [26] and Hess [15] studies. Age structure share of 15–64 years has a positive impact on sustainable development in Table 4, which indicates the correctness of our second hypothesis that APL has a positive impact on SD. Apart from that, when we employ the interaction term of moderating variable, APL*RI showed a positive effect on SD in Tables 4 and 5, all of which are in line with the findings of Hess [15].

However, after employing the interaction of health expenditure*RI, the findings of age structure became negative, as suggested by Dietz, Neumayer [34] in their study of 115 countries and 18 years’ data. Therefore, the findings suggest that every person needs to retain his/her current living standards after retirement or old age from a social point of view, although this would not be feasible, given the current saving rates. As a result, the population requires government assistance through a safety net that places a burden on future generations. The initial distribution of social capital is calculated by 25-year-old population average education years. The population’s age structure, expressed in dependency pressures, can also affect the capacity to save from a given national income. An increase in the ratio of the population under the age of 15 (youth dependents) to the total population, or to the population aged 15 to 64 (net producers), will continue to demand a higher proportion of income for the children’s current social welfare expenditure (education, healthcare, food, and clothing), which is counted as consumption expenditure, vis-a-vis the national income. In line with the life cycle theory of consumption, an increase in the proportion of the dependent population will reduce the national saving rate, as a higher proportion of the population moves into the dissolving years, with increasing elderly healthcare expenses.

Health expenditure per capita (HSPC) has a positive and statistically significant impact on sustainable development, which proves our alternate hypothesis 3, by addressing the third scientific question of the study. These results in Table 3 support the findings of Cheung and Padieu [39], Kofi Boachie, Ramu [4], Sahnoun [40], and Leon, Jimenez [42] studies. Further, after employing the interaction term of HSPC*RI, results unveiled a significant positive impact in Table 6, which are in line with the conclusions of Obere, Muthoga [43], Wang and Selina [44] and Garofoli [45] studies.

Other socio-economic determinants, i.e., governance composite, documented the positive contribution of governance practices towards the sustainable development of the sample countries. As suggested by the past findings of Rajkumar and Swaroop [46] and Stojanovic, Ateljevic [30] about sound governance systems, our sample exhibited a positive influence on both direct and moderating channels in Table 3. The findings indicate that the BRI countries’ governance system significantly contributes to their sustainable development. Population size (PS) has a statistically positive impact both on direct and moderating channels, and these findings are consistent with those of [27, 51]. However, the e-government development index showed a significant impact on SD at significance level in both the empirical equations employed, i.e., first, SD and its determinants, and second, SD and its determinants with moderating variables of RI interaction term, but statistically antagonistic. These outcomes indicate that the e-government system in Belt and Road countries are weak, because the concept of e-government is relatively new and needs significant improvement to implement e-government practices which would lead to a sustainable development path in a country, as internet usage and technology-friendly environment rise over the years [47, 48]. Government size and globalization negatively influence sustainable development in BRI countries. Overall findings indicate that the BRI region is at the progressive stage; though it contributes to achieving sustainable development, it can be more effective by improving the full potential of regional integration among BRI economies.

## 5. Conclusion

This study investigates the effect of age structure share (APL), human capital development (HD), and health expenditure per capita (HSPC) on sustainable development and empirically examines the moderating role of regional integration. The other socio-economic factors include governance composite, e-government development index (EGDI), population size (PS), government size (GS), and globalization index (GI). The study sample includes 64 countries of the Belt & Road Initiative (BRI) with a strongly balanced panel dataset from 2003 to 2018.The pair-wise correlation showed that human development, age structure, health expenditure per capita, governance composite, population size, and e-government have a positive association with sustainable development, while globalization and government size are negatively correlated with it. Two-step system GMM was employed based on Roodman [74] accepted standards and results are further confirmed through robustness check with D-K standard-error regression.

The direct-effect outcomes are mixed and findings reveal that human development, health expenditure, age structure share, governance index, and population size improve sustainable development in BR region, whereas e-government and globalization negatively affect it. The moderating channel of regional integration (RI)-interactions variable of human development with regional integration, and health expenditure with regional integration- enhance sustainable development in the selected region. In contrast, the moderating effect of age structure with regional integration decreases the sustainable development. Other socio-economic determinants such as governance and population size have a positive influence on sustainable development, while government size and globalization hinder the same in BRI countries. Moreover, the authors found a weak e-government system in Belt and Road countries.

The findings of the current research have important policy implications for balanced and sustainable growth. The study suggests that everybody needs to retain their current living standards and health facilities after retirement or old age, from a social perspective. However, this would not be feasible, given the current saving rates. As a result, extensive populations require government assistance in providing healthcare and other basic facilities through a safety net that places a burden on future generations. In line with the life cycle theory of consumption, an increase in the populations without proportionate income will reduce the national saving rate, leading to an unstable sustainable development path. A higher proportion of the population moves into the dissolving years and increases elderly healthcare expenses. The youth and elderly burdens of dependency tend to be reversed, with the former declining during the fertility transition and the latter rising with a replacement fertility approach. Population and urbanization play a crucial role in increasing the demand for healthcare and energy in BRI countries. Governments should take preventive measures to slow down the growth of population urbanization and provide facilities through technology to improve the living standards and healthcare in local areas. BRI governments should also implement proposed UN, WHO and government level population restraints, as rapidly growing populations increase human development and healthcare expenditure, waste and water pollution, which affect sustainable development.

The impact of the BRI initiative on sustainable development in the countries of the Himalayan Region BRI is mainly going through the heart of the Himalayas Region, which will open the opportunity of the establishment of man-made structures and trade, commerce, and business. This will also lead to human settlement, and sustainable development in the countries inhibited to date. Such incidences will hamper the natural balance of the area and negatively affect the flora and fauna of the region because of rapid growth in BRI projects. Affecting natural balance currently gains attention from the practitioner and governments, aiming to invest more in green and renewable energy, which will help to improve environmental quality. However, Governments and concerned practitioners need to focus more on the balanced utilization of natural resources by protecting a friendly environment and harming future generations’ needs.

In the field of public health and human development, immense opportunities are offered by the BRI, involving multiple countries for partnership and collective actions to fight globalization-related emerging pandemics, infectious or chronic diseases, and outbreaks of potential threats to both health information management and laboratory information management systems. Worldwide geo-economics and sustainable development path may improve due to the strengthening of the health system for public health initiatives. By allocating resources for socially responsible industries, making investments in social and green government projects, and restricting eco-unfriendly technology and projects (taxes and penalties), regional integration can be an essential tool for sustainable development in BRI countries. Therefore, in the presence of BRI multi-dimensional regional integration, governmental policymakers of BRI countries need to emphasize and lead towards the emerging e-governance paradigm and innovations in the institutions. This will promote and enhance the institutions’ quality and public facilities that enable accountability, transparency, inclusiveness, effectiveness, and sustainable development. As multi-dimensional regional integration is a dynamic process that expands mutually beneficial dimensions of sustainable development (i.e., social, environmental, economic, and technological) activities of neighboring economies, the coordinated policies follow the common goals and shared prosperity.

The net forest loss projections represent only timber values in sustainable development measures and disregard all external and non-timber benefits associated with standing forests; thus, it may add more value to perfection. Though the public expenditures on education include sustainable development (adjusted net savings), a more comprehensive measure would be to add private spending on education and research and development. Private spending on education would add value to the country’s human capital stock, whereas research and development expenditure could extend the basic knowledge incorporated in sustainable development (adjusted net savings). Future studies on this novel concept can be conducted by adding more social, economic, and environmental determinants; social determinants can include income inequalities, household consumptions, unemployment, and urbanization, while environmental determinants may comprise energy efficiency, natural resource rent and environmental quality and economic determinants may consist of financial development and income per capita in BRI countries by considering untapped multi-dimensional regional integration index and institutional quality.

## Supporting information

S1 Appendix (DOCX)
