# Supplementary material for: Nexus of regional integration, socioeconomic determinants and sustainable development in belt and road initiative countries
Source: PLoS One. 2021 Jul 9;16(7):e0254298. doi: 10.1371/journal.pone.0254298 (PMC8270197; doi:10.1371/journal.pone.0254298)
Supplement: S1 Appendix — (DOCX) [file pone.0254298.s001.docx]

Appendix A. Sample countries along the Belt and Road

| Region and Country |
| --- |
| **East Asia:** China, Mongolia; **Southeast Asia:** Brunei, Cambodia, Indonesia, Laos, Malaysia, Myanmar, Philippines, Singapore, Thailand, and Vietnam. (Timor-Leste is the part of one belt road but not included in the sample due to unavailability of complete sample data); **Central Asia:** Kazakhstan, Kyrgyzstan, Tajikistan, (Turkmenistan, and Uzbekistan are the part of one belt road but not included in the sample due to unavailability of complete sample data); **South Asia**: Bangladesh, Bhutan, India, Nepal, Pakistan, Sri Lanka (Afghanistan, and Maldives are the part of one belt road but not included in the sample due to unavailability of complete sample data)  **The Middle East:** Bahrain, Iraq, Israel, Jordan, Kuwait, Lebanon, Oman, Qatar, Saudi Arabia, Syria and Egypt (Egypt also linked with northeast Africa), (Iran, Palestine, United Arab Emirates and Yemen are the part of one belt road but not included in the sample due to unavailability of complete sample data)  **Africa:** North Africa (Algeria , North Africa); West Africa (Gambia, The, Burkina Faso, Mali, Senegal); East Africa (Djibouti, Ethiopia, Mauritius, Rwanda, Kenya); Southern Africa (South Africa)  **Europe:** Albania, Armenia, Azerbaijan, Belarus, Bulgaria, Croatia, Czech Republic, Estonia, Georgia, Hungary, Latvia, Lithuania, Macedonia, Moldova, Poland, Romania, Russia, Serbia, Slovakia, Slovenia, Turkey (also some part in western Asia), Ukraine (Bosnia and Herzegovina, and Montenegro are the part of one belt road but not included in the sample due to unavailability of complete sample data) |

*Source.* Industrial Cooperation between Countries along the Belt and Road, China International Trade Institute. The countries are grouped based on the World Bank's classification by region

Appendix B. Variables Data Description and Measurement

| **Indicator** | Data Description, Measurement, and followed studies | Data Website |
| --- | --- | --- |
| **Dependent Variable** | | |
| Sustainable Development | $ANSR= \frac{NNS+CSE-\sum R_{n, i}-CD}{\mathrm{GNI}}$  Where, ANS = Adjusted Net Saving Rate, NNS = Net National Savings, CSE = expenditure on education, Rn,i = Rent from depletion of natural capital I (mineral depletion + energy depletion + net forest depletion), CD = Damages from carbon dioxide and particulate emissions, GNI = Gross National Income at Market Prices. ANSR is the fraction of GNI and found by dividing adjusted net savings (ANS) with GNI.  Where, Net national savings (NNS) = (Gross national saving − consumption of fixed capital).  Staff of World bank based on the Changing Wealth of Nations 2018: Building a Sustainable Future described sources and methods in ([64](#_ENREF_64)). Sustainable development measured through ANS (adjusted net saving) proxy followed from ([15-17](#_ENREF_15), [28](#_ENREF_28)). | WDI, World Bank |
| **Independent Variable** | | |
| Human Development (HD) | Human Development (HD) index based on four indicators life expectancy, Per Capita gross national income, schooling mean years, and schooling expected years.  Determinant adopted by the ([15](#_ENREF_15)). | United Nations Development Program; Global Economy Website |
| The average share of the population of ages, APL) | APL (population total percentage average between the ages of 15 and 64 years). APL also called population age structure. Adopted by ([15](#_ENREF_15)). Population share of 65 and older years increased tend to reduce the national saving rate. Population which counts all residents based on the de facto definition. | WDI, World Bank |
| Health Expenditure Per Capita | Health spending per capita measures in USD based on expenditures on current health, including healthcare services and goods disbursed during each year. | The World Bank |
| **Moderating Variable - Interaction Term** | | |
| Regional Integration | RI is used to know the moderating impact on the relationship between the Human Development, Age Structure and Health Expenditure Per Capita determinants and the adjusted net saving rate. Measure through Dummy Variable 1 and 0. Regional Integration dummy variable reflects the One belt region, having a value one after initiative periods and zero for pre-periods.  For each firm-year observation, RI is ranked throughout a period that ranges from 0 to 1 in scale ([81](#_ENREF_81)). | Authors Estimations |
| **Other socio-economic factors** | | |
| Governance Composite | The governance composite is based on word development six indicators rule of law, control of corruption, regulatory quality, political stability & absence of terrorism, government effectiveness, and voice & accountability.  Governance composite is a composite of each country from 2003 to 2018 based on the PCA command in Stata.  World Bank Governance indicators developed by ([16](#_ENREF_16), [30](#_ENREF_30), [82](#_ENREF_82)). | WDI |
| E-Government  Development | There are three most noteworthy components of e-government, i.e.^,^ first is the scope & quality of online services (Online Service Index, OSI), second is inherent social capital index and third is development status of telecommunication infrastructure (Telecommunication Infrastructure Index, TII). Therefore, EGDI is a weighted average of normalized scores of the aforementioned components. A study conducted by ([47](#_ENREF_47)). | UN E-Government Knowledge database |
| Government Size | Government total spending as a percentage of GDP excluding government military expenditures was used as the proxy of total size and followed by ([52](#_ENREF_52), [53](#_ENREF_53)). | World Bank |
| Population size | Population in millions based on the de facto definition counting legal status or citizenship residents and values are the mid-year estimates. Proxy followed from ([27](#_ENREF_27), [51](#_ENREF_51)). | United Nations Population Division |
| Globalization Index | Based social, political, and economic dimensions of globalization concerned on 0 to 100 points of higher values denote greater globalization.  Where, social globalization based on personal contacts (international telecom traffic, degree of tourism, transfers, foreign population, and number of international letters), information flows (number of internet users, the share of households with a television set, and trade in newspapers), and cultural proximity (trade-in books and number of McDonald's restaurants and Ikea located in a country). Political globalization based on the number of high commissions and embassies in a country; the country is a member of international organizations number; UN peace missions as well a country participated in number; and between two more stated treaties signed number. Economic globalization based on economic actual flows (trade data, portfolio investment, and FDI) and trade and capital restrictions (mean tariff rates, hidden import barriers, index of capital controls, and taxes on international trade as a share of current revenue). Adopted and recommended by ([83](#_ENREF_83), [84](#_ENREF_84)) | The Swiss Institute of Technology in Zurich; Global Economy Website |

Appendix C. Results of Stationary (Unit Root) and Westerlund Cointegration Tests

| 1. Unit Root (Stationary) Tests | | | | | | |
| --- | --- | --- | --- | --- | --- | --- |
| **Variables**  **and Decision** | **Lags** | **Levin, Lin & Chu t*** | **Im, Pesaran and Shin W-stat** | **ADF - Fisher Chi-square** | **PP - Fisher Chi-square** | **Decision** |
| SD (I0) | At Level | -6.7828*** | -3.7640*** | 0.0000*** | 6.0841*** | (I0) |
|  | At First Diff | -6.4729*** | 0.0001*** | 18.2046*** | 80.97*** |  |
| HDI (I0) | At Level | -18.9462*** | -6.6050*** | 24.8886 *** | 28.28*** | (I0) |
|  | At First Diff | -14.4959*** | 0.0008*** | 18.3441*** | 89.35*** |  |
| APL (I0) | **At Level** | -7.4985*** | 3.4932 | 25.2839*** | 28.8092*** | (I0) |
|  | At First Diff | -5.0733*** | 0.3366 | 17.1813*** | 19.1542*** |  |
| HSPC (I0) | At Level | -8.1318 *** | -1.8120** | 16.1770*** | 1.4352* | (I0) |
|  | At First Diff | -8.8524 *** | -2.2296** | 16.2306*** | 1.5815* |  |
| Governance  (I1) | At Level | -3.4803*** | -0.2344 | 4.6937*** | 2.2479** (demean) | (I1) |
|  | At First Diff | -4.2625*** | -0.5877 | 8.6801*** | 2.1849** (demean) |  |
| EGDI (I1) | At Level | -3.3291*** (demean) | 8.6844 | 17.2269*** | 28.28*** | (I1) |
|  | At First Diff | -8.1759*** (demean) | 4.359 | 4.6937*** | 89.35*** |  |
| GS (I0) | At Level | -7.2718*** | -2.1518** | 8.6801*** | 4.2024*** | (I0) |
|  | At First Diff | -4.9869*** | -1.3032* | 15.1154*** | 4.5789*** |  |
| PS(I0) | At Level | 19.1373 | 32.1232 | 12.2706*** | 12.1717*** | (I0) |
|  | At First Diff | -6.0109*** | 4.9374 | 12.6511*** | 9.8955*** |  |
| GI | At Level | -19.2400*** | -12.2278*** | 33.9651*** | 25.3210*** | (I0) |
|  | At First Diff | -16.3817*** | -9.0870*** | 0.0000*** | 28.9925*** |  |
| 1. Westerlund test for Cointegration (AR parameter: Panel specific) | | | | | | |
| Dependent | Independent | Indicator | Statistic | p-value | Decision | |
| SD | HDI APL HSPC GOC EGDI PS GI | Variance ratio | -2.4335 | 0.0075 | Ha: Panels are cointegrated  -2.4335*** | |
| SD | HDI APL HSPC GOC EGDI PS GS | Variance ratio | -2.4173 | 0.0078 | Ha: Panels are cointegrated  -2.4173*** | |

*Note.* *** p<0.01, ** p<0.05, * p<0.1 indicate p-value significance at 1%, 5%, and 10% levels, respectively.

*Note.* In Westerlund Cointegration Tests used applied two case determine best understating as in Panel specific cannot include more than 7 variables therefore we make two batch.

*Note.* Number of panels = 64, Number of periods = 16

Appendix D. Robustness Check: Driscoll-Kraay Standard Errors Regression

| Variables | (1) | (2) |
| --- | --- | --- |
|  | Robust Direct Model | |
| Sustainable Development | DK Pooled OLS | DK Fixed effect |
| Human Development (HD) | 4.814***  (4.830) | 24.072  (14.739) |
| Age Structure Share (APL) | 0.659  (0.052) | -0.202  (0.165) |
| Health Expenditure Per Capita (HSPC) | 0.006***  (0.000) | 0.001  (0.001) |
| Governance Composite (GOV) | 3.220***  (0.349) | 0.655  (0.696) |
| E-Government (EGDI) | -6.566  (5.120) | -11.679***  (2.968) |
| Government Size (GS) | -0.439***  (0.033) | -0.712***  (0.115) |
| Population Size (PS) | 0.009***  (0.000) | -0.006  (0.010) |
| Globalization Index (GI) | -0.389***  (0.036) | -0.018  (0.088) |
| Constant | -4.500**  (2.258) | 24.795***  (10.713) |
| R-squared | 0.2773 | 0.0872 |
| Root MSE | 10.1873 | - |
| maximum lag | 2 | 2 |
| F( 8, 15) | 4963.51*** | 162.06*** |
| Number of groups | 64 | 64 |
| Number of Obs. | 1024 | 1024 |

*Note.* Standard errors in parentheses, *** p<0.01, ** p<0.05, * p<0.1 indicate significance at 1%, 5%, and 10% levels, respectively, Used Stata xtscc command— ([76](#_ENREF_76)).

Appendix E. Robustness Check: Driscoll-Kraay Standard Errors Regression

| Variables | (1) | (2) | (3) | (4) | (5) | (6) |
| --- | --- | --- | --- | --- | --- | --- |
|  | Robust Indirect Model (HDI*RI) | | Robust Indirect Model (APL*RI) | | Robust Indirect Model (HSPC*RI) | |
| Sustainable Development | DK Pooled OLS | DK Fixed effect | DK Pooled OLS | DK Fixed effect | DK Pooled OLS | DK Fixed effect |
| Human Development (HD) | 4.274  (4.491) | 36.102*  (17.725) | 4.086  (4.846) | 31.661*  (16.564) | 4.589  (0.364) | 37.296*  (19.246) |
| Age Structure Share (APL) | 0.658***  (0.051) | -0.250  (0.181) | 0.674***  (0.048) | -0.239  (0.167) | 0.656***  (0.051) | -0.243  (0.166) |
| Health Expenditure (HSPC) | 0.006***  (0.000) | 0.001  (0.001) | 0.006***  (0.000) | 0.002  (0.001) | 0.006***  (0.001) | 0.001  (0.002) |
| Governance Composite (GOV) | 3.218***  (0.343) | 0.715  (0.684) | 3.206***  (0.347) | 0.618  (0.696) | 3.214***  (0.339) | 0.747  (0.706) |
| E-Government (EGDI) | -6.299  (5.426) | -9.945***  (3.170) | -5.636  (5.429) | -9.056**  (3.219) | -5.985  (5.353) | -10.047***  (3.347) |
| Government Size (GS) | -0.436***  (0.034) | -0.685***  (0.117) | -0.437***  (0.033) | -0.671***  (0.121) | -0.437***  (0.033) | -0.688***  (0.118) |
| Population Size (PS) | 0.009***  (0.000) | -0.006  (0.010) | 0.009***  (0.000) | -0.005  (0.010) | 0.009***  (0.000) | -0.006  (0.010) |
| Globalization Index (GI) | -0.388***  (0.037) | -0.027  (0.088) | -0.392***  (0.037) | -0.016  (0.084) | -0.390***  (0.036) | -0.027  (0.088) |
| Regional Integration (RI) | -1.387  (1.257) | -1.263  (1.181) | 2.337  (1.969) | 3.231  (3.447) | -0.352  (0.528) | -1.249***  (0.349) |
| HD*RI | 1.519**  (1.799) | 0.269  (1.863) |  |  |  |  |
| APL*RI |  |  | -0.041*  (0.034) | -0.065  (0.056) |  |  |
| HSPC*RI |  |  |  |  | 0.000  (0.001) | 0.000  (0.000) |
| Constant | -4.147*  (2.177) | 19.311*  (10.166) | -5.138**  (2.305) | 20.093*  (9.689) | -4.282*  (2.324) | 18.256*  (9.415) |
| R-squared | 0.2775 | 0.0916 | 0.2776 | 0.0928 | 0.2775 | 0.0918 |
| Root MSE | 10.1958 | - | 10.1955 | - | 10.1962 | - |
| maximum lag | 2 | 2 | 2 | 2 | 2 | 2 |
| F( 8, 15) | 11610.34*** | 633.11*** | 5839.75*** | 586.00*** | 4285.09*** | 491.78** |
| Number of groups | 64 | 64 | 64 | 64 | 64 | 64 |
| Number of Obs. | 1024 | 1024 | 1024 | 1024 | 1024 | 1024 |

*Note.* Standard errors in parentheses, *** p<0.01, ** p<0.05, * p<0.1 indicate significance at 1%, 5%, and 10% levels, respectively, Used Stata xtscc command— ([76](#_ENREF_76)).
